# Genomic Identification and Expression Analysis of Regulator of Chromosome Condensation 1-Domain Protein Family in Maize

**DOI:** 10.3390/ijms252111437

**Published:** 2024-10-24

**Authors:** Rui Liu, Tian Ma, Yu Li, Xiongbiao Lei, Hongjing Ji, Hewei Du, Jianhua Zhang, Shi-Kai Cao

**Affiliations:** 1 School of Life Science, Yangtze University, Jingzhou 434025, China; liuxiaoshuang_6@163.com (R.L.); 19164047603@163.com (T.M.); tinashecool@163.com (Y.L.); leixionbiao627@163.com (X.L.); jjing0105@163.com (H.J.); duhewei666@163.com (H.D.); 2 Department of Biology, Hong Kong Baptist University, Hong Kong, China; 3 State Key Laboratory of Agrobiotechnology, The Chinese University of Hong Kong, Hong Kong, China

**Keywords:** RCC1-containing protein, bioinformatic analysis, abiotic stress, maize (*Zea mays*)

## Abstract

Abiotic stress affects the growth and development of maize (*Zea mays*). The regulator of chromosome condensation 1 (RCC1)-containing proteins (RCPs) plays crucial roles in plant growth and development and response to abiotic stresses. However, a comprehensive analysis of the maize RCP family has not been reported in detail. This study presents a systematic bioinformatics analysis of the ZmRCP family, identifying a total of 30 members distributed across nine chromosomes. The physicochemical properties and *cis*-acting elements in the promoters of *ZmRCP* members are predicted. The results of subcellular localization showed that ZmRCP3 and ZmRCP10 are targeted to mitochondria and ZmRCP2 is localized in the nucleus. A heatmap of expression levels among family members under abiotic stress conditions revealed varying degrees of induced expression, and the expression levels of 10 *ZmRCP* members were quantified using RT-qPCR under abiotic stress and plant hormone treatments. The results showed that *ZmRCP* members exhibit induced or inhibited responses to these abiotic stresses and plant hormones. These results contribute to a better understanding of the evolutionary history and potential role of the ZmRCP family in mediating responses to abiotic stress in maize.

## 1. Introduction

Maize is an important cereal crop that often suffers from abiotic stresses such as drought, high temperature, low temperature, and salinity during its growth and development, leading to severe yield reduction [1]. During long periods of evolution, organisms have evolved a range of mechanisms to cope with a variety of stresses, such as UV protection and osmo-protection mechanisms [2,3]. Regulator of chromosome condensation 1 (RCC1)-containing proteins (RCPs) have been reported to mediate diverse biological processes, including these abiotic stress responses [4,5,6].

Proteins that contain one or more RCC1/RCC1-like domains/repeats are classified as RCC1 proteins/RCPs [7]. Each repeat contains 51-68 amino acid residues and adopts a seven-bladed β-propeller fold [4,8]. RCP members are commonly found in eukaryotic cells. In humans, RCC1 is a key cell cycle regulatory factor involved in chromosome binding, nuclear cytoplasmic transport, and cell cycle control [8]. In animals, RCC1 is involved in many biological processes coupled with Ran in the nucleus, where RanGDP can produce RanGTP under the action of RCC1 [9,10].

In plants, RCPs have been identified in several species, including rice (*Oryza sativa*), maize (*Zea mays*), cotton (*Gossypium Hirsutum*), *Populus euphratica*, and *Arabidopsis thaliana* [5,6,11,12,13,14]. In Arabidopsis, 24 putative RCP family members have been identified. Six AtRCP proteins have been characterized, including UVR8 (ultraviolet receptor 8), tolerant to chilling and freezing (TCF1), RCC1/UVR8/GEF like 1 (RUG1), RUG2, RUG3, and sensitive to ABA 1 (SAB1) [5,11,12,15,16,17,18]. Under UV-B irradiation, UVR8 can dissociate from homologous dimers into monomers, which can interact with COP1 and continue to accumulate in the nucleus, triggering the UV-B cascade reaction, so that the plant activates the UV protection mechanism [3,19]. RUG1 encodes bilirubin deaminase (PBGD), which is an enzyme in the tetrapyrrole biosynthesis pathway that produces chlorophyll, heme, and so on. Deficiency of *RUG1* has been shown to lead to leaf damage, abnormal plant development, and down-regulated expression of auxin-related genes [20]. RUG2 encodes a dual localization protein, which localizes in both mitochondria and chloroplasts, and its mutation exhibits pale green leaves, growth retardation, and a reduced number of mesophyll cells [16]. During seed germination and post-germination growth, mutation of *RUG3* enhances the responses to ABA, suggesting that RUG3 as a negative regulatory factor mediates the ABA signaling pathway in Arabidopsis [17]. Arabidopsis SAB1 is also an RCP member as a negative regulator of *ABI5* (*abscisic acid-insensitive 5*); it functions upstream of *ABI5* and regulates promoter binding activity, its stability, and histone-methylation-mediated gene silencing of *ABI5* post-germination [11]. Moreover, Arabidopsis TCF1 encodes a glycosylphosphatidylinositol anchored protein that mediates a plant-specific freezing-resistant transcription program by regulating lignin biosynthesis independently of the CBF pathway [5]. These cases indicate that RCP members are involved in the response to abiotic stresses in Arabidopsis.

In addition, rice *Root Length Regulator 4* (*OsRLR4*) encodes an RCP protein that affects root elongation by negatively regulating RAM (root apical meristem) activity [13]. Overexpression of *Populus euphratica UVR8* (*PeUVR8*) in the Arabidopsis *uvr8* mutant rescued its phenotype and overexpression of *PeUVR8* in wild-type Arabidopsis enhanced anthocyanin accumulation and inhibited hypocotyl elongation, indicating that PeUVR8 functions in controlling plant photomorphogenesis [14]. In maize, several RCP family members have been reported. For example, DEK47 is responsible for splicing four introns of mitochondrial *nad2* in maize; therefore, it is critical for the assembly and activity of mitochondrial complex I and seed development [21]. The down-regulated expression of *ZmRCC1-1* causes cytological changes, such as altering grain orientation and affecting cell cycle progression. *ZmRCC1-2* and *ZmRCC1-3* neutralize the changes caused by *ZmRCC1-1* down-regulation, returning chromosome function to a normal state [22]. Although RCP members have been studied for over thirty years, there is still a lack of systematic identification and an analysis of the RCP family in maize.

In this study, we identified a total of 30 RCP members in maize through a systematic bioinformatics analysis. A comprehensive analysis of the ZmRCP family, including physicochemical properties, a phylogenetic tree, and subcellular localization, is performed. The expression analysis of *ZmRCP* genes were determined by RT-qPCR under abiotic stresses and plant hormones treatments. These data provide theoretical support and optional gene resources for future research on the biological functions of the ZmRCP family and the cultivation of stress-resistant maize varieties.

## 2. Results

### 2.1. Identification and Prediction of Protein Physicochemical Properties

**Figure 1:**
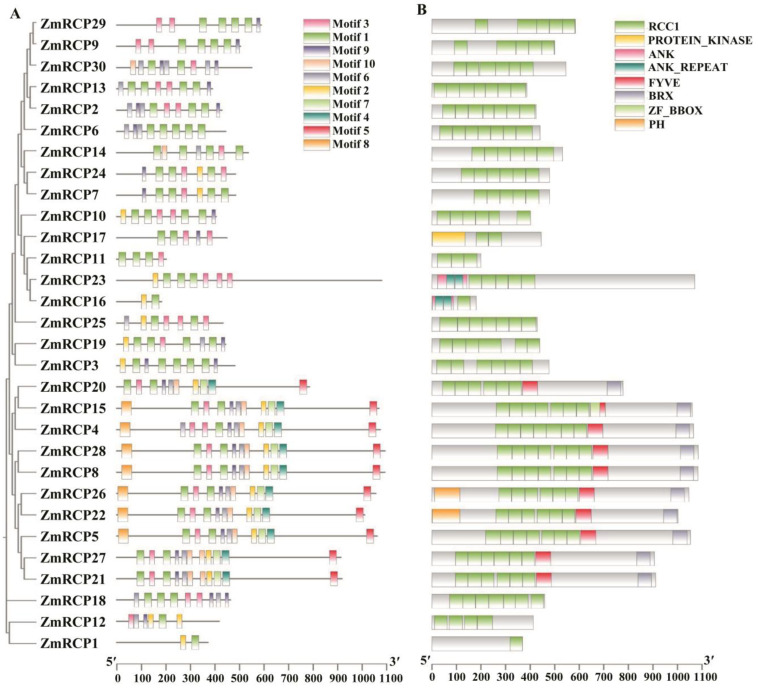
Prediction of conserved motifs and domains of ZmRCP family members in maize. (**A**) The phylogenetic relationship (left panel) and conserved motifs (right panel) of ZmRCP members are listed. (**B**) Prediction of conserved domains of ZmRCP members.

To identify the RCP family members in maize, we operated the homologous alignment using the Arabidopsis RCP proteins using bidirectional BLASTp from NCBI. After domain validation, a total of 30 maize RCP family members were identified (Table 1, Figure 1). The number of amino acid residues (aa) in the ZmRCP family ranges from 181 to 1092. The molecular weight of ZmRCP family proteins ranges from 19,246.71 Da to 117,959.71 Da. The isoelectric point is between 4.95 and 9.84; most are alkaline proteins. The instability index of ZmRCP family proteins ranges from 6.81 to 49.16, and 19 members have an instability index lower than 40, indicating their stability. The hydrophilicity index ranges from −0.609 to −0.01.

### 2.2. Phylogenetic Relationship and Collinearity Analysis

**Figure 2:**
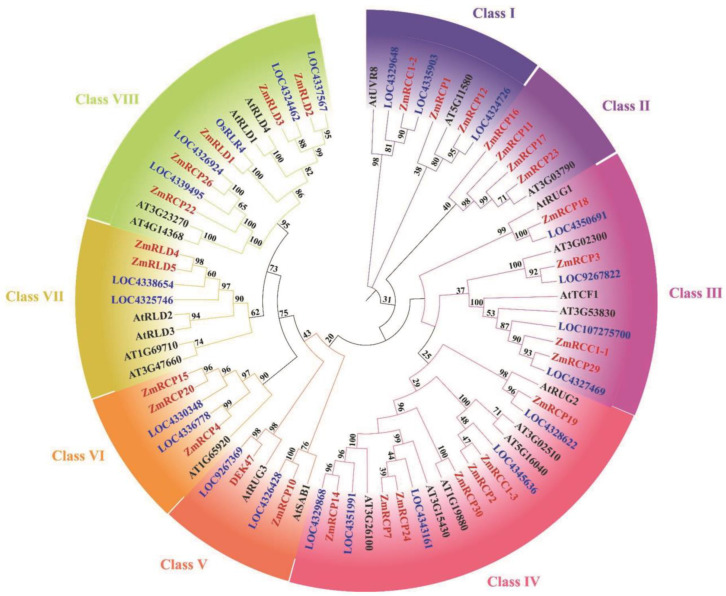
Phylogenetic tree of RCP family members among maize, rice, and Arabidopsis. Bootstrap values are marked at the branches. Different groups are marked with different colors.

In order to explore the specific phylogenetic relationship of RCP members in maize and other species, we constructed a phylogenetic tree of RCP proteins in three species, including *Zea mays*, *Arabidopsis thaliana*, and *Oryza sativa*. After domain validation of the rice protein sequence downloaded from NCBI, it was found that there are 22 members of the same domain in rice. There are 30 members in maize, 24 members in Arabidopsis, and 23 members in rice, indicating that the number of RCP family members is relatively conservative with no significant expansion (Figure 2). After constructing the tree using the adjacency method, all RCP family members in these three species were clustered into eight classes based on their genetic relationships, from Class I to VIII. In maize, there are two members in Class V, three members in Class I, VI, and VII, four members in Class II and III, five members in Class VIII, and seven members in Class IV (Figure 2).

To further understand the evolutionary relationship of the *RCP* gene family in different species, we selected three plants for a species collinearity analysis, *Zea mays*, *Oryza sativa*, and *Arabidopsis thaliana*. The results showed that there are two *RCP* genes in *Zea mays* that are collinear with *Arabidopsis thaliana* (Figure S1). However, there are 22 *RCP* genes in maize that are collinear with *Oryza sativa*. Among them, *ZmRCP9* and *ZmRCP29* are collinear with both *Arabidopsis thaliana* and *Oryza sativa*. Compared with dicotyledonous plants like *Arabidopsis thaliana*, more *RCP* genes in *Zea mays* show collinearity with monocotyledonous plants like *Oryza sativa*, indicating that *Zea mays* and *Oryza sativa*, both of which are monocotyledonous plants, have a higher homology of RCP members. Most *RCP* genes originated from the ancestors of monocotyledonous plants, while a small number of genes may originate from dicotyledonous plants.

Collinearity analysis is a technique used to identify homologous protein sequences in the genome that are arranged in almost the same order at different positions in order to further reveal the genetic relationships between or within species of these proteins. The results of an intra-species collinearity analysis in maize showed that there were eight fragment duplication events on chromosomes 1, 2, 3, 4, 5, 7, and 8, involving a total of 12 genes (Figure S2). Based on the results of the evolutionary tree, we found that two genes with collinearity are classified in the same class (Figure 2). For example, *ZmRCP2* and *ZmRCP13*/*ZmRCC1-3* both belong to the Class IV subfamily, indicating that their gene structures and conserved domains are similar, which further supports the rationality of evolutionary tree grouping. The members of the *RCP* gene family have a certain degree of conservation and many gene duplications, which not only play an important role in the survival process of maize but also provide possibilities for the diversification of gene functions and gene evolution.

### 2.3. Conserved Motifs, Domains, and Gene Structure Analysis

In order to study the genetic structural changes of the RCP family genes in maize, their gene structures were analyzed. The diagram of gene structure shows that all members of the maize RCP family contain different quantities of introns, and most members contain untranslated regions at the 5′ and/or 3′ ends except for three members (Figure S3). The numbers of introns of RCP members contained are from 2 to 15.

A motif is commonly referred to as a super secondary structure. This structure is formed by the aggregation of secondary structural units of proteins, and the motif plays an important role in the function and structure of proteins. The prediction of motifs of RCP family members showed that the *ZmRCP* gene family in maize contains a total of 10 motifs, with ZmRCP1 and ZmRCP16 having only two motifs (Figure 1A). The vast majority of members contain motif 1 and motif 3, except for ZmRCP1, ZmRCP3, ZmRCP6, and ZmRCP16 (Figure 1A).

The conserved domains of the maize *RCP* gene family were analyzed using the MEME tool of Tbtools. The prediction results of conserved domains showed that all the ZmRCP members have the RCC1 domain, and the amounts of the RCC1 domain are from 1 to 7 (Figure 1B). In addition, there are the PROTEIN_KINASE, ANK (Ankyrin), ANK-REAPEAT, FYVE, BRX (BREVIS RADIX domain), ZF_BBOX (B-box type zinc finger), and PH (Pleckstrin Homology) domains. Among them, there are members that are relatively close in evolutionary relationships, such as ZmRCP29 and ZmRCP9 and ZmRCP13 and ZmRCP2 (Figure 1B).

### 2.4. Distribution of ZmRCP Family Members on Chromosomes

**Figure 3:**
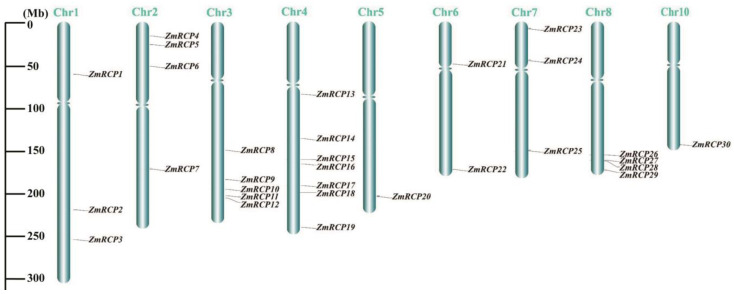
Distribution of *ZmRCP* family members on chromosomes (Chr). Gray lines indicate the location of the genes on the chromosomes, and the length of the chromosomes is shown on the left scale.

After obtaining the chromosomal location information of *ZmRCP* family members from the NCBI and MaizeGDB databases, TBtools software II was used to visualize it. The diagram showed that the 30 members of the *ZmRCP* family are distributed on nine chromosomes (Chr), with 1 member on Chr5 and Chr10; 2 members on Chr6; 3 members on each of Chr1 and Chr7; 4 members on each of Chr2 and Chr8; 5 members on Chr3; and 7 members on Chr4 (Figure 3). Most of the genes are distributed at the ends of the chromosome.

### 2.5. Subcellular Localization of ZmRCP Family Members

**Figure 4:**
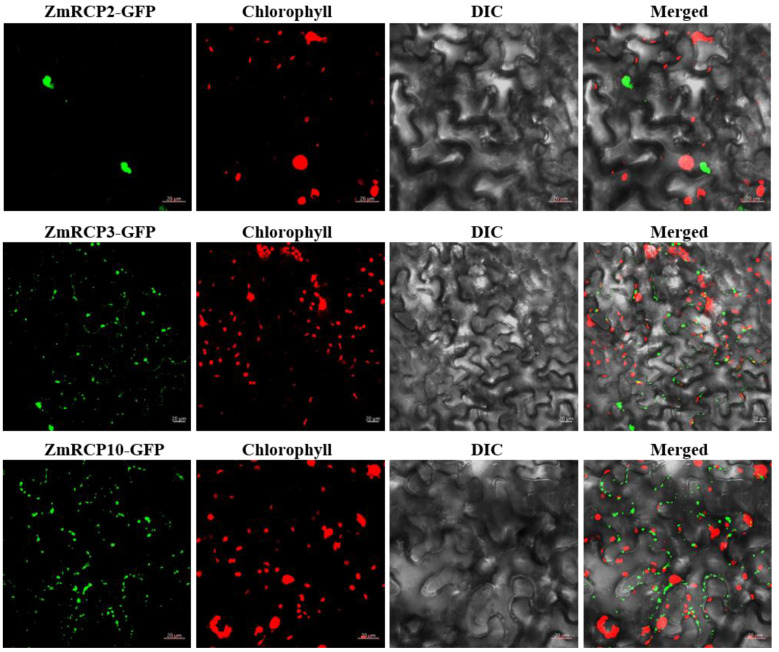
The subcellular localization of ZmRCP2, ZmRCP3, and ZmRCP10 in tobacco leaf epidermal cells. Fluorescence signals from GFP (green) and chlorophyll (red) were imaged under a confocal microscope. Scale bars = 20 µm.

In order to understand the subcellular compartments where RCP family members are located, we conducted a subcellular localization prediction (Table 1). The results indicate that there are 22 ZmRCPs localized in the nucleus and four members localized in both the cell wall and nucleus. To verify the accuracy of subcellular localization prediction, we selected three family members for subcellular localization observation, ZmRCP2, 3, and 10. The resulting constructs ZmRCP2-GFP, ZmRCP3-GFP, and ZmRCP10-GFP were transfected to *Nicotiana benthamiana* leaves via *Agrobacterium*-mediated methods. Fluorescence signals were imaged using fluorescence confocal microscopy. The results showed that the green fluorescence signals of ZmRCP2-GFP were observed in the nucleus, whereas ZmRCP3-GFP and ZmRCP10-GFP were detected in dots that were not merged with the chloroplast autofluorescence signals (Figure 4), indicating that ZmRCP3-GFP and ZmRCP10 are targeted to mitochondria.

### 2.6. Prediction of Cis-Elements in the Promoters

**Figure 5:**
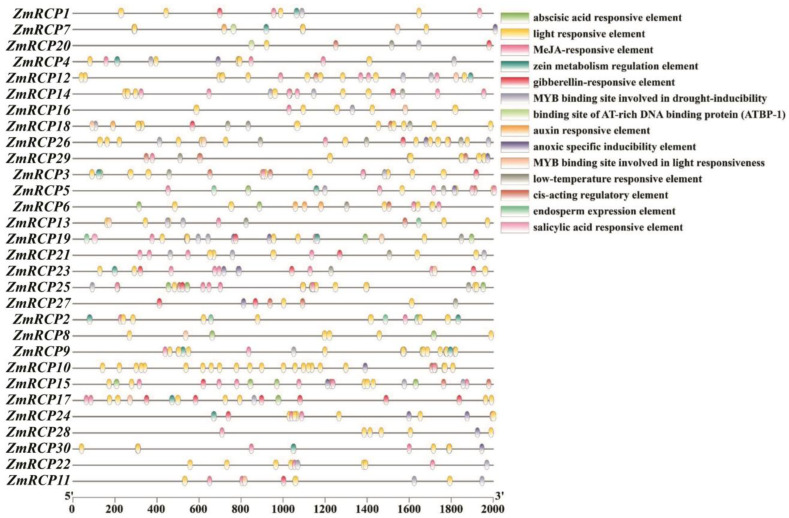
Prediction of *cis*-elements in promoters of 30 *ZmRCP* family members. Various colored boxes indicate different *cis*-elements that correspond to each colored box shown on the right side of the picture, and promoter lengths (base pairs) are shown on the scale below.

The 2000 bp sequences upstream of the start codons of 30 *ZmRCP* genes were extracted as the promoter regions used for *cis*-element prediction (Figure 5). The predicted *cis*-acting elements can be roughly divided into three categories: abiotic stress, plant hormones, and the growth and development process of maize. Abiotic-stress-responsive elements include drought, low-temperature, anoxic, and light-responsive elements. Plant-hormone-responsive elements include abscisic acid, MeJA, gibberellin, salicylic acid, and auxin-inductive or -responsive elements. The *cis*-acting elements during the growth and development of maize include the zein metabolism regulation element and endosperm expression element. All *ZmRCP* family members’ promoter regions contain light-responsive elements, and the majority of *ZmRCP* family members’ promoters contain multiple light-responsive elements. A total of 14 *ZmRCP* member promoter regions contain low-temperature responsive elements, and 22 *ZmRCP* gene promoter regions contain a MYB binding site involved in drought-inducibility elements. These prediction results indicate that the *ZmRCP* family may play an important regulatory role in the growth and development of and response to abiotic stress and plant hormones. The prediction of *cis*-acting elements can provide ideas for future research on related genes.

### 2.7. Analysis of Expression Patterns under Different Tissues and Stresses

**Figure 6:**
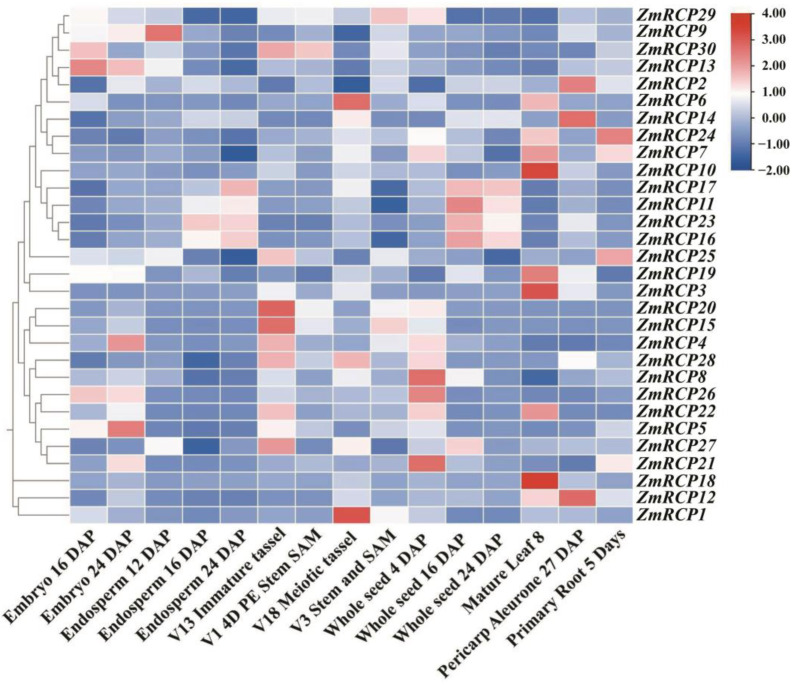
Expression pattern of 30 *ZmRCP* family members in different developmental stages and tissues. The different colors indicate the expression levels of the *ZmRCP* genes in maize. The expression data were downloaded from qTeller in MaizeGDB (https://qteller.maizegdb.org/genes_by_name_B73v5.php (accessed on 27 June 2024)) and the heatmap was generated using Tbtools software.

In plants, gene expression in different tissues is often closely related to their functions. By analyzing transcriptome data from different tissues or developmental stages of plants, the expression levels of gene family members in a certain tissue or developmental stage can be determined, leading to speculation that these genes may play important roles in plant growth and development. The results of an expression pattern heatmap of *ZmRCP* members showed that the relative expression levels of genes in the red area are higher, while those in the blue area indicate lower levels (Figure 6). A total of 24 *ZmRCP* genes have relatively higher expression levels in embryos, endosperms, seeds, or the pericarp aleurone than other genes, indicating that these genes may function in the development of embryos, endosperms, seeds, or seed germination. As shown in Figure 6, *ZmRCP30*, *29*, and *15* have higher expression levels in SAM. Z*mRCP6*, *24*, *7*, *10*, *19*, and *3* have higher expression levels in mature leaf 8 than others. The expression of *ZmRCP24*, *7*, and *25* is higher than other genes in primary roots at day 5 (different days after pollination), indicating that these three genes may play a role in root development.

To investigate the stress response of *ZmRCP* family members under abiotic stresses, we analyzed the RNA-seq data online of *ZmRCP* members under different abiotic stresses, including heat, cold, salt, UV, and drought stress. The results showed that the expression levels of *ZmRCP29*, *9*, and *3* were induced under heat stress; the expression of Z*mRCP30*, *7*, *10*, *17*, *11*, *23*, *16*, and *20* was induced by cold stress; the expression of Z*mRCP29, 9, 13, 25, 3, 5, 27, 18, 12,* and *1* was induced by salt stress; and the expression of Z*mRCP30, 13, 17, 23, 16, 25, 20, 15, 4, 28, 26, 5, 27,* and *21* was induced by UV stress (Figure S4A). In addition, the expression of Z*mRCP29, 20, 14, 17, 11, 23, 16, 19, 3, 20, 15, 4, 8, 27,* and *18* was induced by a mild water deficit (24 h). Under a severe water deficit, the expression of *ZmRCP29, 9, 14, 7, 10, 17, 11, 23, 16, 3, 20, 4, 28, 26, 22, 5, 27, 21, 18*, and *12* was induced (24 h) (Figure S4B). These data suggest that ZmRCP family members possibly play important roles in the response to abiotic stresses.

**Figure 7:**
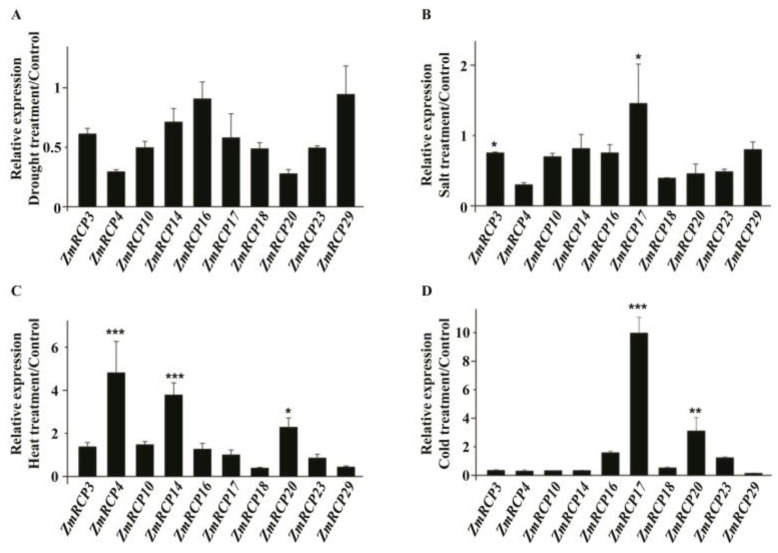
Expression levels of 10 *ZmRCP* members under abiotic stress treatments. Expression levels of 10 *ZmRCP* members were detected by RT-qPCR under drought (**A**), salt (**B**), heat (**C**), and cold (**D**) treatments. *ZmActin* (GRMZM2G126010) was used as the reference gene. Data are means (±SE) of three biological replicates. The comparison groups of Student’s *t*-test are treatment and control. Asterisks indicate significant differences between means calculated with Student’s *t*-test. * *p* < 0.05; ** *p* < 0.01; *** *p* < 0.001.

To further study the potential role of *ZmRCPs*, we performed RT-qPCR to detect the expression levels of 10 family members (*ZmRCP3*, *4*, *10*, *14*, *16*, *17*, *18*, *20*, *23*, and *29*) under different abiotic stresses and plant hormone treatments, which were induced by abiotic stresses based on RNA-Seq data online (Figure S4). The results showed that the transcript of these 10 *ZmRCP* members was decreased under drought stress at 6 h (Figure 7A), while the transcript of *ZmRCP17* was increased under salt stress (Figure 7B). *ZmRCP4*, *14*, and *20* were remarkably induced under heat stress (Figure 7C), whereas *ZmRCP17* and *20* were significantly induced under clod stress (Figure 7D). These results suggest that *ZmRCP* members differentially respond to drought, salt, heat, and cold treatments.

**Figure 8:**
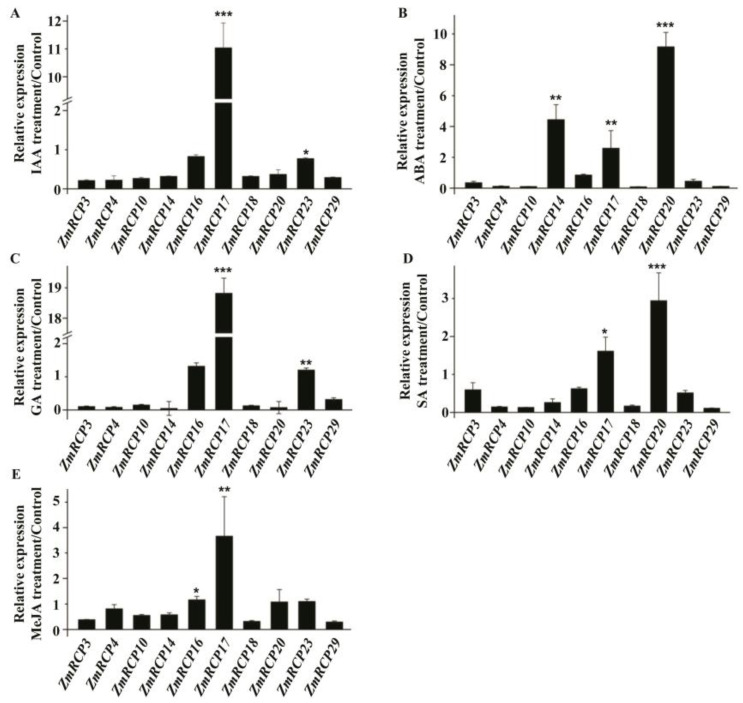
Expression levels of 10 *ZmRCP* members under different hormone treatments. Expression levels of 10 *ZmRCP* members were detected by RT-qPCR under IAA (**A**), ABA (**B**), GA (**C**), SA (**D**), and MeJA (**E**) treatments. *ZmActin* (GRMZM2G126010) was used as the reference gene. Data are means (±SE) of three biological replicates. The comparison groups of Student’s *t*-test are treatment and control. Asterisks indicate significant differences between means calculated with Student’s *t*-test. * *p* < 0.05; ** *p* < 0.01; *** *p* < 0.001.

In addition, *ZmRCP17* showed induced expression under IAA, GA, and MeJA treatment (Figure 8A,C,E). *ZmRCP14*, *17*, and *20* showed induced expression under ABA treatment (Figure 8B), while *ZmRCP17* and *20* showed induced expression under GA treatment (Figure 8E). These results suggest that *ZmRCP* members respond to different hormone treatments.

### 2.8. Prediction of Protein Interaction Network

In order to understand the potential interacting proteins of RCP gene family members, we used a website to predict the possible interacting proteins of RCP family members and then drew the diagram of the protein interaction network. There are a total of 37 proteins that possibly interact with the RCP family members, with the majority of RCP family members interacting with ATG7 (Figure S5). The ATG7 protein is an autophagy-related protein that allows cells to degrade damaged tissues through autophagy while also providing energy to help plants resist external stress and adverse factors [23,24]. In addition, other possible interacting proteins predicted include PSAH (photosystem I reaction center subunit VI) and TIDP3014 (GTP binding nucleoprotein), etc., most of which are related to stress and plant growth and development [25,26]. These results suggest that the ZmRCP family members may participate in plant growth and development processes and respond to abiotic stress through protein–protein interactions.

**Figure 9:**
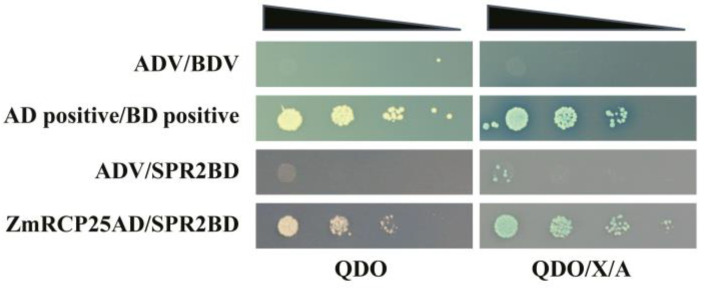
ZmRCP25 interacts with SPR2 in yeast two-hybrid system. Yeast two-hybrid assay was performed to determine interactions between ZmRCP25 and SPR2. These constructs were co-transfected into Y2H Gold strain and QDO (SD/−Ade/-His/−Leu/−Trp) medium with added X-a-gal and AbA (QDO/X/A) medium to reveal protein–protein interactions. AD: GAL4 activation domain; BD: GAL4 DNA binding domain.

A previous study reported that ZmRCP25 (ZmDEK47) is required for the splicing of four introns of mitochondrial *nad2* transcript and seed development. The small PPR protein 2 (SPR2) is required for the splicing of 15 mitochondrial group II introns in maize, including the 4 introns of *nad2* [27], indicating that the functions of these two splicing factors are overlapped with each other. To test whether these two proteins interact with each other, we performed a yeast two-hybrid assay. The results showed that ZmRCP25AD/SPR2BD can grow on QDO or QDO/X/A medium (Figure 9), indicating that ZmRCP25 interacts with SPR2 in the yeast system.

## 3. Discussion

In this study, a phylogenetic tree of RCP family members was constructed among maize, rice, and Arabidopsis (Figure 2). The results showed that there are slightly more members of the RCP family in maize than in rice and Arabidopsis, indicating that the number of members of the RCP family has slightly increased during species evolution and that its functions may be more diverse in maize. Regarding the RCC1-conserved domain, members of the RCP family typically consist of six or seven RCC1 domains [12]. Most members in maize still retained this characteristic, but some members had a reduced number of RCC1 domains, or even only one RCC1 domain remained (Figure 1B). This may be because some conservative structural domains are lost when dealing with changes in the environment or other situations. Gene duplication is important for biological evolution, as it can promote the production of new adaptive functions in plants and expand gene families [28]. In the maize *RCP* gene family, a total of eight fragment duplication events were identified (Figure S2). These repeated fragments may enable maize to adapt to complex environments.

Previous studies reported that RCP members function in various aspects of plant growth and development. For example, the RCC1 domain protein ZmRCP25/DEK47 is required for the splicing of mitochondrial *nad2* introns and kernel development in maize [21]. The small PPR protein SPR2 is also required for the splicing of *nad2* introns and kernel development in maize [27]. Interestingly, ZmRCP25 interacted with SPR2 in the yeast two-hybrid assay (Figure 9), implying that ZmRCP25/SPR2 may form a protein complex in intron splicing. The RCC1-containing protein RUG3 is involved in the splicing of mitochondrial *nad2* intron 2 and 3 in Arabidopsis [12]. AtRUG3 and DEK47 cluster into a branch in Class V (Figure 2), which are both required for the intron splicing of mitochondrial *nad2*, indicating they have a functional homology. Similarly, ZmRCP18 is in Class III with AtRUG1, which is related to chlorophyll synthesis [20], implying that ZmRCP18 may be related to chlorophyll synthesis. Moreover, ZmRCP19, 2, 30, 24, 7, and 14 are in the Class IV, as are AtRUG2 and ZmRCC1-3 (Figure 2). AtRUG2 maintains normal transcriptional function in chloroplasts and mitochondria and ZmRCC1-3 and ZmRCC1-2 attenuate the defects of the cell cycle caused by ZmRCC1-1 dysfunction [16,22], implying that these ZmRCPs may be involved in certain functions in chloroplasts and mitochondria or play a role in cell cycle regulation. Because of the importance of chloroplast photosynthesis and mitochondrial oxidative respiration, loss of these ZmRCPs may cause severe defects in plant growth and development and even embryo lethality in maize.

Several RCP members were reported to be involved in response to abiotic stresses, such as responding to UV stress, cold stress, drought stress, heat stress, and salt stress [5,29,30]. For instance, AtUVR8 is involved in chromatin condensation and plays a role in the UV-B signaling pathway, inducing flavonoid biosynthesis [31]. AtUVR8 can activate a protection mechanism to cope with UV stress. Under drought stress, salt stress, or cold stress, the expression level of the *AtUVR8* gene increased compared to the corresponding sensitive and wild-type plants [3,19,29]. The RCC1-domain protein TCF1 can improve plant cold tolerance by reducing lignin accumulation under low-temperature conditions in Arabidopsis and soybeans [5,30]. In our data, the phylogenetic tree analysis showed that ZmRCC1-1 and ZmRCP29 are clustered in the same branch as AtTCF1, and ZmRCC1-2 is clustered in the same branch as AtUVR8 (Figure 2). Meanwhile, the prediction of *cis*-acting elements shows that promoter regions of multiple *ZmRCP* members contain low-temperature-responsive elements and a MYB binding site involved in drought-inducibility elements (Figure 5). The heatmap of gene expression under abiotic stress shows that the expression levels of most ZmRCP members increase under UV stress (Figure S4A). Moreover, the expression of several *ZmRCP* members was induced under different abiotic stresses (Figure 6 and Figure S4A,B). These results suggest that the *ZmRCP* family may play important regulatory roles in the response to abiotic stresses in maize.

In previous reports, RCP family members have also been shown to respond to plant hormones such as ABA and auxin [5,11]. ABA and auxin are important plant hormones that regulate plant growth, development, and stress response. ABA plays a crucial role in various physiological processes of plants, such as stomatal closure, the accumulation of epidermal wax, leaf senescence, bud dormancy, seed germination, osmotic regulation, and growth inhibition [31]. The RCC1 protein SAB1 is involved in ABA signaling and affects the stability of ABI5 by initiating complex mechanisms, thereby negatively regulating the expression of *ABI5* genes [11]. RCC1 proteins AtRLD1 and AtRLD4 change the gravitropism of roots by altering the distribution of auxin [32]. The prediction of *cis*-acting elements shows that promoters of many ZmRCP members contain elements responding to plant hormones, including auxin, abscisic acid, MeJA, gibberellin, etc. (Figure 5). In addition, RT-qPCR results showed that the expression of *ZmRCP14*, *17*, and *20* was remarkably induced under different plant hormone treatments (Figure 7). These data suggest that *ZmRCP* members are involved in the response to plant hormones in maize.

Based on the above results, it is speculated that the *ZmRCP* gene may play a role in the response to abiotic stress and plant hormones, particularly in drought stress and cold stress. Although the potential functions of ZmRCP family members may be speculated based on the above analysis, whether these members truly participate in these functions still needs to be experimentally proven.

## 4. Materials and Methods

### 4.1. Materials and Treatments

Maize inbred line B73 seedlings grown to three leaves were used for abiotic stress and hormone treatments, and RNA was extracted for subsequent RT-qPCR. Seedlings were subjected to drought, salt, or control treatments with 20% (m/v) PEG-6000, 200 mM NaCl, or ddH_2_O for 6h, respectively. Cold, high-temperature, and control treatments were carried out in a light incubator at a low temperature of 4 °C, a high temperature of 42 °C, and a control temperature of 25 °C for 24 h, respectively. Hormone treatments were carried out with sterile water (ddH_2_O) or abscisic acid (ABA), auxin (IAA), salicylic acid (SA), methyl jasmonate (MeJA), and gibberellin (GA) at concentrations of 100 μmol·L^−1^, and the hormone solutions were used to soak roots for 4 h. Each of the above groups was planted with 3 cups of sand-cultured maize seedlings, and 5 seeds were sown in each cup.

### 4.2. Identification of RCP Family Members

The genome and annotation files of maize were downloaded from Ensembl Plants (http://plants.ensembl.org/info/data/ftp/index.html (accessed on 16 February 2024)). The 24 *Arabidopsis thaliana* RCP family members were searched, and their protein sequences were downloaded from the Arabidopsis TAIR (https://www.arabidopsis.org/ (accessed on 16 February 2024)) website. The extraction of the protein sequences of maize was carried out using TBtools software II [33,34], and the protein sequences of 24 *Arabidopsis thaliana* RCP family members were used as candidate family members using BLASTp bidirectional BLAST from NCBI (https://blast.ncbi.nlm.nih.gov/ (accessed on 16 February 2024)). The InterPro (https://www.ebi.ac.uk/interpro/ (accessed on 18 February 2024)) website was used to detect the RCP structural domain, and the ones that did not contain this conserved domain were removed, which finally determined the maize *RCP* gene family member list.

### 4.3. Construction of Phylogenetic Tree

The rice RCP members were obtained through a homologous blast. The RCP protein sequences of *Arabidopsis thaliana*, *Oryza sativa*, and *Zea mays* were imported, and a sequence alignment was performed by applying the ClustalW algorithm of MEGA7.0 software, choosing the neighbor-joining method, and setting the step value (Bootstrap) to 1000 to construct the multi-sequence phylogenetic tree [35]. The phylogenetic tree was uploaded to iTOL (https://itol.embl.de/itol.cgi (accessed on 26 February 2024)) to beautify and classify it.

### 4.4. Collinearity Analysis

The intra-species and inter-species collinearity analyses of maize RCP members were performed using McScanX, and the collinearity diagrams were drawn using the built-in Circos function in Tbtools software [33,34].

### 4.5. Analysis of Cis-Acting Elements in the Promoters

The 2000 bp promoter sequences upstream the start codon of each of the maize RCP family members were obtained from the official maize database MaizeGDB (https://maizegdb.org/ (accessed on 19 May 2024)), the *cis*-acting elements of each promoter of the maize RCP family members were predicted using the online tool PlantCARE (https://bioinformatics.psb.ugent.be/webtools/plantcare/html/ (accessed on 20 May 2024)), and the drawing/diagram was completed using TBtools software [33,34].

### 4.6. Prediction of Protein Physicochemical Properties

The protein molecular weight, instability index, average hydropathicity, isoelectric point, and number of amino acid residues of RCP family members were analyzed by uploading each identified protein sequence to the ExPASy ProtParam official website (https://web.expasy.org/protparam/ (accessed on 11 March 2024)). The subcellular localization of each family member was predicted using CELLO (http://cello.life.nctu.edu.tw/ (accessed on 12 March 2024)), wolf psort (https://wolfpsort.hgc.jp/ (accessed on 12 March 2024)), and Cell-Ploc (http://www.csbio.sjtu.edu.cn/bioinf/Cell-PLoc-2/ (accessed on 12 March 2024)).

### 4.7. Gene Structure, Conserved Motif, and Structural Domain Analysis

The information about the length of open reading frames and the number of exons of maize RCP family members was obtained from the MaizeGDB (https://maizegdb.org/ (accessed on 25 March 2024)). The conserved motifs were predicted by the MEME online tool (https://memesuite.org/meme/tools/meme (accessed on 26 March 2024)), and the number of conserved motifs was set as 10. The conserved domains of the maize RCP family members were further obtained by using the CD-search tool on the official website of NCBI (https://www.ncbi.nlm.nih.gov/ (accessed on 27 March 2024)). Diagrams of gene structure, protein conserved motifs, and conserved domains were visualized using the Gene Structure View function in TBtools software [33,34].

### 4.8. Location on Chromosome

The diagram of chromosome localization was obtained by uploading GFF3 files and gene IDs in the Gene Location Visualize from GTF/GFF tool of TBtools [33,34].

### 4.9. Expression Pattern Analysis

The expression data of ZmRCP family members under abiotic stresses and in different tissues were downloaded from qTeller in MaizeGDB. The clustering analysis was performed and the expression heatmaps of maize RCP family members were generated by using Tbtools software [33,34].

### 4.10. Subcellular Localization

The full-length cDNAs of *ZmRCP-2, 3*, and *10* were cloned into the pSuper1300-GFP vector and fused to the N-terminal of GFP (Green Fluorescence Protein). The constructs were transformed into tobacco leaves through *Agrobacterium EHA105*-mediated transformation as described previously [27]. The subcellular localization of the target proteins was preliminarily determined where the was green fluorescence signal observed.

### 4.11. Analysis of Protein Interaction Network Prediction

The interaction relationships corresponding to maize RCP proteins were extracted directly from the STRING (https://string-db.org/ (accessed on 29 July 2024)) database and the PlantSPEAD database [36]. The protein interaction network was constructed by inputting the protein interaction information in Cystoscope v3.10.1 software.

### 4.12. RT-qPCR

Total RNA was extracted from 0.2 g of leaves from seedlings after treatment with different hormones or abiotic stresses using the RNA easy Isolation Reagent kit (Vazyme Biotech Co., Ltd., Nanjing, China). Reverse transcription was conducted after DNA digestion using a HiFi Script cDNA Synthesis Kit (Vazyme Biotech Co., Ltd., Nanjing, China) using 1 μg total RNA as the template. RT-qPCR was performed with a CFX96 Real Time PCR instrument (Bio-Rad, Hercules, CA, USA) and an SYBR Green I Master PCR kit (Roche, Basel, Switzerland) using *ZmActin* (GRMZM2G126010) as the internal reference gene. Three independent biological and technical replicates were performed, and the relative expression level of the gene was calculated using the formula 2^-ΔΔCt^ [37]. The primers used in this study are in Supplementary Table S1.

### 4.13. Yeast Two-Hybrid Assay

The full-length cDNA of ZmRCP25 and SPR2 was cloned into pGADT7 and pGBKT7, respectively. ZmRCP25-pGADT7 and SPR2-pGBKT7 were co-transformed into the yeast strain *Y2H Gold*. The growth conditions of the co-transformants were imaged after being inoculated onto Minimal Media Quadruple Dropout (QDO, SD-Ade/-His/-Leu/-Trp) plates for 4 days as described previously [27].

## 5. Conclusions

In this study, 30 *ZmRCP* members were identified and analyzed in maize through a systematic bioinformatics analysis. We performed a physicochemical property analysis, a phylogenetic tree analysis, *cis*-acting elements prediction, and subcellular localization of ZmRCP. Several *ZmRCP* members exhibited induced or inhibited responses to abiotic stresses and plant hormones. These results contribute to a better understanding of the evolutionary history and potential role of the ZmRCP family in mediating responses to abiotic stresses in maize.

## Figures and Tables

**Table 1:** Prediction of physicochemical properties of the maize RCP family members.

Gene ID	Protein Name	Subcellular Localization	Protein Length/aa	Molecular Mass/Da	Theoretical pI	Instability Index	Grand Average of Hydropathicity
*Zm00001eb016900*	ZmRCP1	Chloroplast	371	41,019.26	4.95	35.26	−0.308
*Zm00001eb041760*	ZmRCP2	Nucleus	427	45,901.78	8.38	28.27	−0.349
*Zm00001eb049950*	ZmRCP3	Nucleus	480	51,477.95	5.94	30.7	−0.206
*Zm00001eb072860*	ZmRCP4	Nucleus	1073	118,737.65	9.00	37.37	−0.468
*Zm00001eb075990*	ZmRCP5/ZmRLD1	Nucleus	1060	115,768.8	8.47	41.97	−0.458
*Zm00001eb082500*	ZmRCP6/ZmRCC1-2	Nucleus	443	47,158.43	5.38	37.24	−0.346
*Zm00001eb096520*	ZmRCP7	Cell wall, nucleus	483	51,229.18	6.62	27.16	−0.010
*Zm00001eb140790*	ZmRCP8/ZmRLD4	Chloroplast, nucleus	1091	118,047.68	8.89	43.98	−0.448
*Zm00001eb148070*	ZmRCP9/ZmRCC1-1	Nucleus	503	51,936.8	5.43	45.05	−0.263
*Zm00001eb151590*	ZmRCP10	Nucleus	404	43,284.09	5.59	29.71	−0.230
*Zm00001eb153510*	ZmRCP11	Cell wall, nucleus	200	21,328.17	9.84	6.81	−0.177
*Zm00001eb154440*	ZmRCP12	Nucleus	416	45,496.38	5.21	43.89	−0.181
*Zm00001eb179510*	ZmRCP13/ZmRCC1-3	Nucleus	389	41,874.04	6.01	28.34	−0.351
*Zm00001eb183880*	ZmRCP14	Cell wall, mitochondrion	535	57,269.85	5.65	32.71	−0.093
*Zm00001eb187440*	ZmRCP15	Nucleus	1067	117,523.14	8.91	43.04	−0.475
*Zm00001eb188870*	ZmRCP16	Nucleus	181	19,246.71	8.54	19.79	−0.118
*Zm00001eb196450*	ZmRCP17	Nucleus	448	49,266.91	9.51	24.39	−0.031
*Zm00001eb197640*	ZmRCP18	Nucleus	462	49,028.32	6.40	43.18	−0.102
*Zm00001eb205920*	ZmRCP19	Nucleus	442	46,469.45	5.70	36.51	−0.034
*Zm00001eb250930*	ZmRCP20	Nucleus	784	86,289.9	8.85	39.96	−0.506
*Zm00001eb267290*	ZmRCP21/ZmRLD2	Nucleus	917	99,449.7	8.51	35.57	−0.400
*Zm00001eb294600*	ZmRCP22	Nucleus	1009	109,869.18	8.83	42.92	−0.407
*Zm00001eb300770*	ZmRCP23	Nucleus	1078	116,042.22	9.12	42.32	−0.447
*Zm00001eb306630*	ZmRCP24	Cell wall, nucleus	483	51,361.1	6.52	29.82	−0.048
*Zm00001eb320310*	ZmRCP25/DEK47	mitochondria	432	45,433.14	6.49	36.49	−0.153
*Zm00001eb360990*	ZmRCP26	Nucleus	1054	115,204.69	8.71	39.45	−0.482
*Zm00001eb362420*	ZmRCP27/ZmRLD3	Nucleus	912	98,988.94	8.70	46.22	−0.470
*Zm00001eb362840*	ZmRCP28/ZmRLD5	Nucleus	1092	117,959.71	8.95	43.31	−0.445
*Zm00001eb367810*	ZmRCP29	Nucleus	588	61,993.36	6.22	49.16	−0.350
*Zm00001eb432950*	ZmRCP30	Cell wall, nucleus	549	58,115.71	8.42	38.59	−0.609

## Data Availability

Data are contained within the article and Supplementary Materials.

## Supplementary Materials

The following supporting information can be downloaded at https://www.mdpi.com/article/10.3390/ijms252111437/s1.
